# Performance Analysis of Two-Way Satellite Multi-Terrestrial Relay Networks with Hardware Impairments

**DOI:** 10.3390/s18051574

**Published:** 2018-05-15

**Authors:** Kefeng Guo, Kang An, Bangning Zhang, Daoxing Guo

**Affiliations:** 1 College of Communication Engineering, Army Engineering University of PLA (Its Old Name Is PLA University of Science and Technology), Nanjing 210007, China; guokefeng.cool@163.com (K.G.); bangning_zhang@sina.com (B.Z.); 2 National University of Defense Technology, Nanjing 210016, China; ankang@nuaa.edu.cn

**Keywords:** satellite-terrestrial networks, two-way terrestrial relays, opportunistic relay selection scheme, hardware impairments (HIs)

## Abstract

In this paper, we investigate the performance of a two-way hybrid satellite multi-terrestrial cooperative network with hardware impairments (HIs). Particularly, opportunistic relay selection scheme is employed in the considered network, which can substantially enhance the spectral efficiency and spatial diversity gain. By considering both the amplify-and-forward (AF) and decode-and-forward (DF) relay protocols, the closed-form expressions of the outage probability (OP) and throughput for the system are derived. Furthermore, in order to observe the effects of HIs level and the number of terrestrial relays on the system performance at high signal-to-noise ratios (SNRs), the asymptotic expressions of the system performance are also derived. Finally, computer results are presented to reveal the correctness of the analytical results.

## 1. Introduction

Satellite communication (Satcom) has become an outgrowth of the continuing demand for higher capacity, real-time communication and wider coverage, due to its unique ability to provide seamless connectivity and high data rate [1,2,3]. In addition, Satcom is a more economical solution to provide a reliable and high speed connectivity than deploying other terrestrial networks, especially in some remote and sparsely populated locations [4,5]. However, Satcom systems are prone to the practical masking effect, where the line-of-sight (LOS) communication between the satellite and terrestrial user may be blocked due to obstacles and shadowing.

For the reasons mentioned above, a hybrid satellite-terrestrial cooperative network (HSTCN) has been provided to overcome the disadvantages [6,7,8,9,10,11,12,13,14]. In [7], the authors analyzed the system performance of the satellite-terrestrial networks by using maximal ratio combining (MRC) over shadowed-Rician fading channel. In [8], the authors addressed the problem of amplify-and-forward (AF) relaying in HSTCN, where a masked destination node could receive both the direct transmitted signal and relayed signal from a terrestrial link and the symbol error rate (SER) of the considered system is derived. In [9], the authors proposed the beamforming (BF) and combining scheme for a two-way AF protocol based communication between two multi-antenna earth stations, where the asymptotic expression for the SER of the considered system is also obtained. In [10], the authors analyzed the SER for the HSTCN with AF protocol. Besides, the authors in [11] derived the analytical expression for the ergodic capacity of the HSTCN. In [12], the authors examined the problem of AF based relaying in a hybrid satellite terrestrial link and derived the novel expressions for the SER of the considered system. In [13], the authors investigated the performance of integrated wireless sensor and multi-beam satellite networks under terrestrial interference and derived the closed-form approximations of capacity per beam. In [14], the authors proposed the energy efficient optimal power allocation schemes in the cognitive satellite terrestrial networks for non-real-time and real-time applications and maximized the energy efficiency of the cognitive satellite user.

Multiple terrestrial relays can bring significant system performance growth by increasing the diversity gain of the network. Relay selection scheme can get the balance between the complexity and efficiency. Among the selection schemes, full relay and partial relay selection schemes are two important parts [15,16]. Opportunistic relay selection scheme is the special case of the partial relay selection scheme [17,18]. Until now, several open literatures have incorporated the relay selection scheme into the HSTCN. For example, in [19], the authors derived the exact outage probability of the HSTCN, where a selection scheme of the best relay terminal was performed. In [20], the authors analyzed the multiple terrestrial relay and multiple user HSTCN with max-max selection scheme, where the analytical expression for the outage probability (OP) was derived. In [21], the authors studied the ergodic capacity of the HSTCN with multiple terrestrial relays using full relay selection scheme, where all terrestrial relays cooperate with each other in transmitting the source signal. Furthermore, the authors in [22] analyzed the system performance of the multiple secondary networks in a cognitive HSTCN environment with partial selection scheme.

However, in practice, hardware is not always ideal. Many types of impairments may affect the hardware, for example, phase noise, I/Q imbalance, and high power amplifier nonlinearities [23,24,25,26,27,28,29,30]. In [26,27], the authors studied the outage performance of orthogonal frequency-division multiplexing dual-hop opportunistic AF relaying in the presence of I/Q imbalance (IQI) in all nodes. In [28], the authors investigated the dual-hop AF cooperative systems in the presence of I/Q imbalance. In [29,30], the authors analyzed the impact of I/Q imbalance in full-duplex relay networks. In [31], the authors concluded all the factors and proposed a general model which has been cited by most of the related papers. In [32], the authors studied the impact of hardware impairments (HIs) on the considered two-way relay networks by deriving the analytical expression of the OP. To its regret, they only paid attention to the HIs at relay and neglected the HIs at sources. In [33], the two-way multiple antenna multiple relay networks with HIs were studied and all nodes were considered suffering HIs, and the closed-form expression of the OP for the considered network was also derived. In [34], the authors analyzed the impact of HIs on the multiple relays network with partial relay selection scheme. In [35], the impact of HIs was analyzed based on the cognitive network in the presence of interference. In [36], HIs has been considered as a key problem that needs to be solved in 5G communication networks. In [37], the authors have given the new HIs model, which is the extension of [31]; it is the practical and commonly applied approach in the communication network.

The HIs of the transmitted node not only have an effect on the terrestrial network but they also have an impact on satellite networks. HIs of satellite could reduce the HSTCN performance; it is essential to research the effect of HIs on the Satcom. Until now there is some literature studying the effect of HIs on the Satcom networks, and even HSTCN systems. In [38], the authors first considered the HIs in two-hop Satcom systems and analyzed the effect of HIs on the system performance. The analytical expression of OP for the considered system was derived in shadowed-Rician channel. In [39], the authors derived the analytical expression of the OP for the satellite relay network in the presence of interference and HIs. In [40], the authors studied the OP for the considered networks by means of relay selection scheme without direct link in HIs environment. In [41], the OP was studied for the multiple terrestrial HSTCN with a switch-and-stay combining scheme in the presence of HIs.

However, two-way relay can bring better system performance by increasing the utilization of the spectrum. In two-way networks, the source and destination can receive the signal at the same time with the help of two-way relays. The research for HIs on two-way satellite-terrestrial network is quite limited. We know that only the authors of [42] derived the closed-form expressions of the OP and the throughput for the considered satellite two-way terrestrial networks. Unfortunately, the authors of [42] just considered one terrestrial relay for AF protocol and ignored the performance improvement by the diversity of multiple terrestrial relays.

Motivated by the above discussions, we take the HIs into account and investigate the performance of the two-way hybrid satellite multi-terrestrial cooperative network, where the two-way terrestrial relays are equipped with multiple antennas. Particularly, our main contributions can be summarized as follows:

- Firstly, taking the HIs into account, we propose a framework of two-way hybrid satellite multi-terrestrial cooperative network, where the two-way terrestrial relays are used to assist the signal transmission. Besides, the HIs system model used in this paper is established according to the literatures [37] which is the extension of [31] and the practical and commonly applied approach in the relay communication network.
- Secondly, based on the principle of opportunistic relay selection scheme [17,18], the closed-form expressions for the OP and the throughput of the considered network are derived with AF and decode-and-forward (DF) protocols, which give clear views on the difference between the AF and DF protocol.
- Finally, to gain more sights at high SNRs, the asymptotic OP expressions of the system performance for both the AF and DF protocols are also derived, from which we can know that the HIs level, the number of terrestrial relays and the number of antennas have great effects on the system performance at high SNRs.

The rest of this paper is constructed as follows. The system model and problem formulation is provided in Section 2. In Section 3, the system performance of the considered networks is investigated. In Section 4, computer simulations are provided to verify the correctness of the theoretical results. In Section 5, a brief summary of this paper is provided.

**Notations**: Bold uppercase letters denote matrices and bold lowercase letters denote vectors; ${\left(\cdot \right)}^{H}$ denotes the conjugate transpose, $∥\cdot ∥$ the Euclidean norm of a vector, $\left|\cdot \right|$ the absolute value of a complex scalar; $\exp\left(\cdot \right)$ is the exponential function, $E\left[\cdot \right]$ the expectation operator, $CN\left(a,b\right)$ the complex Gaussian distribution of a random vector a and covariance matrix *b*, $C^{M\times N}$ the M×N dimensional complex positive semidefinite matrix.

## 2. System Model and Problem Formulation

**Figure 1:**
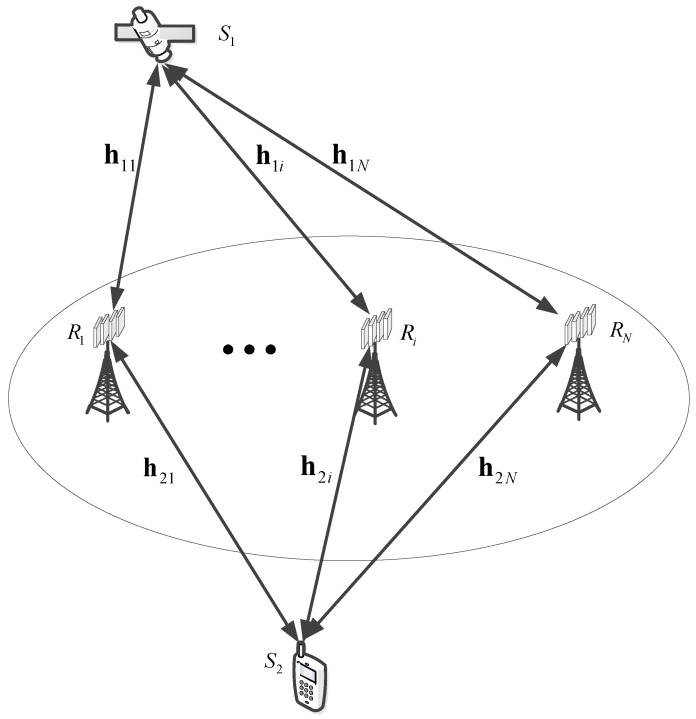
Illustration of the system model.

As provided in Figure 1, this paper studies the two-way hybrid satellite multi-terrestrial communication network, where the satellite and mobile user communicate with each other instead of having the help of multi-two-way terrestrial relays. Due to the heavy fading or huge obstructions, we assume that there is no direct link between the satellite and mobile user (For example, the mobile user is indoors or in the cave, where there is no direct link between the satellite and the mobile user. This assumption has been used in many previous papers [2,3,8,10,11].). In the system, a satellite source node ($S_{1}$), *N* terrestrial nodes (*R*) and a mobile source node ($S_{2}$) are considered. $S_{1}$ and $S_{2}$ are equipped with a single antenna, respectively. *R* is equipped with *M* antennas. The opportunistic relay selection scheme is used to get better system performance. Opportunistic relaying selection scheme is expressed as follows: the system selects the relay link which has the largest signal-to-noise-and-distortion ratio (SNDR) among all the relay links.

It takes two time slots for the communication. In the first time slot, $S_{1}$ and $S_{2}$ transmit the information signals $x_{1}\left(t\right)$ and $x_{2}\left(t\right)$ to the *i*-th *R*, respectively. Hence the received signal at the *i*-th *R* in the first time slot is presented as

(1) $$\begin{array}{c} y_{r}\left(t\right)=w_{i}^{H}h_{1i}\left[\sqrt{k_{1}P_{1}}x_{1}\left(t\right)+\eta_{1}\left(t\right)\right]+w_{i}^{H}h_{2i}\left[\sqrt{k_{2}P_{2}}x_{2}\left(t\right)+\eta_{2}\left(t\right)\right]+w_{i}^{H}n_{i}\left(t\right), \end{array}$$

where $w_{i}^{H}$ is the BF vector at the *i*-th R with $E\left[{∥w_{i}∥}^{2}\right]=1$, $h_{1i}$ the channel coefficient vector for $S_{1}$-the *i*-th R link satisfying shadowed-Rician fading. $k_{1}$ denotes the impairments level at $S_{1}$ satisfying $0\leq k_{1}\leq 1$, where $k_{1}=1$ denotes the ideal hardware. $P_{1}$ is the transmitted power at $S_{1}$, $x_{1}\left(t\right)$ the transmitted signal from $S_{1}$ with $E\left[{\left|x_{1}\left(t\right)\right|}^{2}\right]=1$, $\eta_{1}\left(t\right)$ the distortion noise due to HIs satisfying $\eta_{1}\left(t\right)∼CN\left(0,\left(1-k_{1}\right)P_{1}\right)$. $h_{2i}$ is the channel coefficient vector for $S_{2}$-the *i*-th R obeying Rayleigh fading. $k_{2}$ presents the impairments level at $S_{2}$ with $0\leq k_{2}\leq 1$, $P_{2}$ the transmitted power from $S_{2}$, $x_{2}\left(t\right)$ the signal transmitted from $S_{2}$ obeying $E\left[{\left|x_{2}\left(t\right)\right|}^{2}\right]=1$, $\eta_{2}\left(t\right)$ the distortion noise due to HIs satisfying $\eta_{2}\left(t\right)∼CN\left(0,\left(1-k_{2}\right)P_{2}\right)$, $n_{i}\left(t\right)$ the additive white Gaussian noise (AWGN) at the *i*-th R which is represented as $n_{i}\left(t\right)∼CN\left(0,\delta_{i}^{2}\right)$.

In the second time slot, the *i*-th *R* simultaneously forwards the received signal to $S_{j}$ $\left(j=1,2\right)$. Particularly, we use both AF and DF protocols in this paper. In what follows, without loss of generality, the received signals of $S_{j}$ from the *i*-th *R* for these two protocols are derived, respectively.

### 2.1. AF Protocol

For AF protocol, in the second time slot, the *i*-th R forwards the received signal with a forward gain *G*, then the received signal at $S_{j}$ from the *i*-th R is given by

(2) $$\begin{array}{c} y_{ji}\left(t\right)=w_{i}^{H}h_{ji}\left(\sqrt{k_{3}P_{r}}Gy_{r}\left(t\right)+\eta_{3}\left(t\right)\right)+n_{j}\left(t\right),j\in \left\{1,2\right\}, \end{array}$$

where

(3) $$\begin{array}{c} G=\sqrt{\frac{P_{r}}{{\left|w_{i}^{H}h_{1i}\right|}^{2}P_{1}+{\left|w_{i}^{H}h_{2i}\right|}^{2}P_{2}+\delta_{i}^{2}}}, \end{array}$$

$k_{3}$ represents the impairments level at the *i*-th R, $P_{r}$ the transmitted power of the *i*-th R. $\eta_{3}$ denotes the distortion noise due to HIs with power of $\eta_{3}\left(t\right)∼CN\left(0,\left(1-k_{3}\right)P_{r}\right)$, $n_{j}$ the AWGN at the *j*-th source obeying $n_{j}\left(t\right)∼CN\left(0,\delta_{j}^{2}\right)$.

### 2.2. DF Protocol

For DF protocol, the *i*-th R only forwards the useful signal to $S_{j}$ and ignores the noise. Hence, the received signal at $S_{j}$ is derived as

(4) $$\begin{array}{c} y_{ji}\left(t\right)=w_{i}^{H}h_{ji}\left(\sqrt{k_{3}\left(P_{1}+P_{2}\right)}\left[x_{1}\left(t\right)+x_{2}\left(t\right)\right]+\eta_{3}\left(t\right)\right)+n_{j}\left(t\right). \end{array}$$

## 3. System Performance

In this section, the end-to-end SNDR, the exact and asymptotic closed-form expressions for the OP and the throughput of the considered network with HIs for AF and DF protocols are obtained, respectively. Especially, the opportunistic terrestrial relay selection scheme is applied to the network to get better system performance.

### 3.1. The End-To-End SNDR of the System

In what follows, the final SNDRs of the system for the two considered forward protocols are derived in the following, respectively.

#### 3.1.1. The SNDR for AF Protocol

Now, by taking the *i*-th transmitted link for an example, we first provide the expression of the $y_{1i}\left(t\right)$ which is the signal received by $S_{1}$ from the *i*-th R. By substituting (1) and (3) into (2), $y_{1i}\left(t\right)$ is given by

(5) $$\begin{array}{cc} y_{1i}\left(t\right) & ={\left|w_{i}^{H}h_{1i}\right|}^{2}\sqrt{k_{3}k_{1}P_{1}P_{r}}Gx_{1}\left(t\right)+{\left|w_{i}^{H}h_{1i}\right|}^{2}\sqrt{k_{3}P_{r}}G\eta_{1}\left(t\right)+{∥w_{i}^{H}∥}^{2}h_{1i}h_{2i}\sqrt{k_{3}k_{2}P_{2}P_{r}}Gx_{2}\left(t\right)\\ & +{∥w_{i}^{H}∥}^{2}h_{1i}h_{2i}\sqrt{k_{3}P_{r}}G\eta_{2}\left(t\right)+w_{i}^{H}h_{1i}\sqrt{k_{3}P_{r}}Gw_{i}^{H}n_{1}\left(t\right)+w_{i}^{H}h_{1i}\eta_{3}\left(t\right)+n_{j}\left(t\right). \end{array}$$

As $S_{1}$ wants to distill $x_{2}\left(t\right)$ from $y_{1i}\left(t\right)$, and it knows its own transmitted signal $x_{1}\left(t\right)$ [32], it can perfectly remove the corresponding self-interference term ${\left|w_{i}^{H}h_{1i}\right|}^{2}\sqrt{k_{3}k_{1}P_{1}P_{r}}Gx_{1}\left(t\right)$. Then, the remaining signal at $S_{1}$ for the detection of symbol $y_{1i}\left(t\right)$ is given by

(6) $$\begin{array}{cc} y_{1i}\left(t\right) & ={\left|w_{i}^{H}h_{1i}\right|}^{2}\sqrt{k_{3}P_{r}}G\eta_{1}\left(t\right)+{∥w_{i}^{H}∥}^{2}h_{1i}h_{2i}\sqrt{k_{3}k_{2}P_{2}P_{r}}Gx_{2}\left(t\right)+w_{i}^{H}h_{1i}\eta_{3}\left(t\right)\\ & +w_{i}^{H}h_{1i}\sqrt{k_{3}P_{r}}Gw_{i}^{H}n_{1}\left(t\right)+{∥w_{i}^{H}∥}^{2}h_{1i}h_{2i}\sqrt{k_{3}P_{r}}G\eta_{2}\left(t\right)+n_{j}\left(t\right). \end{array}$$

From (6), we can easily get the SNDR at $S_{1}$ which is given by

(7) $$\begin{array}{c} \gamma_{1i}=\frac{\frac{{\left|w_{i}^{H}h_{1i}\right|}^{2}P_{1}{\left|w_{i}^{H}h_{2i}\right|}^{2}P_{2}}{\delta_{i}^{4}}}{\frac{{\left|w_{i}^{H}h_{1i}\right|}^{2}P_{1}{\left|w_{i}^{H}h_{2i}\right|}^{2}P_{2}}{\delta_{i}^{4}}A_{1}+\frac{{\left({\left|w_{i}^{H}h_{1i}\right|}^{2}P_{1}\right)}^{2}}{\delta_{i}^{4}}B_{1}+\frac{{\left|w_{i}^{H}h_{1i}\right|}^{2}P_{1}}{\delta_{i}^{2}}C_{1}+\frac{{\left|w_{i}^{H}h_{2i}\right|}^{2}P_{2}}{\delta_{i}^{2}}D_{1}+E} \end{array}$$

To get better system performance, MRC and maximum ratio transmission (MRT) technologies are used at the *i*-th R in the received and transmitted slot, respectively. After setting $\lambda_{1i}=\frac{{\left|h_{1i}\right|}^{2}P_{1}}{\delta_{i}^{2}}$ and $\lambda_{2i}=\frac{{\left|h_{2i}\right|}^{2}P_{2}}{\delta_{i}^{2}}$, the SNDR at $S_{1}$ is given by

(8) $$\begin{array}{c} \gamma_{1i}=\frac{\lambda_{1i}\lambda_{2i}}{\lambda_{1i}\lambda_{2i}A_{1}+\lambda_{1i}^{2}B_{1}+\lambda_{1i}C_{1}+\lambda_{2i}D_{1}+E_{1}}, \end{array}$$

where $A_{1}=\frac{1-k_{2}k_{3}}{k_{2}k_{3}}$, $B_{1}=\frac{\delta_{1}^{2}\left(1-k_{1}k_{3}\right)}{\delta_{2}^{2}k_{2}k_{3}}$, $C_{1}=\frac{P_{1}\delta_{1}^{2}+P_{r}\delta_{i}^{2}}{\delta_{2}^{2}P_{r}k_{3}k_{2}}$, $D_{1}=\frac{P_{1}}{P_{r}k_{2}k_{3}}$, and $E_{1}=\frac{P_{1}}{\delta_{1}^{2}\delta_{i}^{2}k_{3}k_{2}P_{r}}$.

With the same method, the SNDR of $y_{2i}\left(t\right)$ is given by

(9) $$\begin{array}{c} \gamma_{2i}=\frac{\lambda_{1i}\lambda_{2i}}{\lambda_{1i}\lambda_{2i}A_{2}+\lambda_{2i}^{2}B_{2}+\lambda_{2i}C_{2}+\lambda_{1i}D_{2}+E_{2}}, \end{array}$$

where $A_{2}=\frac{1-k_{1}k_{3}}{k_{1}k_{3}}$, $B_{2}=\frac{\delta_{2}^{2}\left(1-k_{2}k_{3}\right)}{\delta_{1}^{2}k_{1}k_{3}}$, $C_{2}=\frac{P_{2}\delta_{2}^{2}+P_{r}\delta_{i}^{2}}{\delta_{1}^{2}P_{r}k_{3}k_{1}}$, $D_{2}=\frac{P_{2}}{P_{r}k_{1}k_{3}}$, and $E_{2}=\frac{P_{2}}{\delta_{2}^{2}\delta_{i}^{2}k_{3}k_{2}P_{r}}$.

As opportunistic relay selection scheme is used in the system, hence the final SNDR for AF protocol is given by

(10) $$\begin{array}{c} \gamma_{ae}=\max_{i\in \left\{1,\ldots ,N\right\}}\left\{\min\left(\gamma_{1i},\gamma_{2i}\right)\right\}. \end{array}$$

#### 3.1.2. The SNDR for DF Protocol

Now, we consider the DF protocol, without loss of generality, we also take $S_{1}$ as an example. As mentioned before, MRC is used at the *i*-th R, by recalling (1) and (4), the SNDRs at the *i*-th R and $S_{1}$ for $S_{1}$–$S_{2}$ transmitted link are, respectively, given by

(11) $$\begin{array}{cc} \gamma_{r1i} & =\frac{{\left|h_{2i}\right|}^{2}k_{2}P_{2}}{{\left|h_{1i}\right|}^{2}P_{1}+{\left|h_{2i}\right|}^{2}P_{2}\left(1-k_{2}\right)+\delta_{i}^{2}}\\ & =\frac{\lambda_{2i}}{\lambda_{1i}F_{1}+\lambda_{2i}L_{1}+1}, \end{array}$$

where $F_{1}=\frac{\delta_{1}^{2}}{\delta_{2}^{2}k_{2}}$ and $L_{1}=\frac{1-k_{2}}{k_{2}}$.

(12) $$\begin{array}{cc} \gamma_{r2i} & =\frac{{\left|h_{1i}\right|}^{2}k_{3}P_{2}}{{\left|h_{1i}\right|}^{2}\left(P_{1}+P_{2}\right)\left(1-k_{3}\right)+\delta_{1}^{2}}\\ & =\frac{\lambda_{1}}{\lambda_{1}F_{2}+L_{2}}, \end{array}$$

where $F_{2}=\frac{\left(P_{1}+P_{2}\right)\left(1-k_{3}\right)}{P_{2}k_{3}}$ and $L_{2}=\frac{P_{1}}{k_{3}P_{2}}$.

In the same way, the SNDRs at the *i*-th R and $S_{2}$ for $S_{2}$-$S_{1}$ transmitted link are, respectively, expressed as

(13) $$\begin{array}{cc} \gamma_{r3i} & =\frac{{\left|h_{1i}\right|}^{2}k_{1}P_{1}}{{\left|h_{2i}\right|}^{2}P_{2}+{\left|h_{1i}\right|}^{2}P_{1}\left(1-k_{1}\right)+\delta_{i}^{2}}\\ & =\frac{\lambda_{1i}}{\lambda_{2i}F_{3}+\lambda_{1i}L_{3}+1}, \end{array}$$

where $F_{3}=\frac{\delta_{2}^{2}}{\delta_{1}^{2}k_{1}}$ and $L_{3}=\frac{1-k_{1}}{k_{1}}$.

(14) $$\begin{array}{cc} \gamma_{r4i} & =\frac{{\left|h_{2i}\right|}^{2}k_{3}P_{1}}{{\left|h_{2i}\right|}^{2}\left(P_{1}+P_{2}\right)\left(1-k_{3}\right)+\delta_{2}^{2}}\\ & =\frac{\lambda_{2i}}{\lambda_{2i}F_{4}+L_{4}}, \end{array}$$

where $F_{4}=\frac{\left(P_{1}+P_{2}\right)\left(1-k_{3}\right)}{P_{1}k_{3}}$ and $L_{4}=\frac{P_{2}}{k_{3}P_{1}}$.

The DF protocol is used in the network, hence the corresponding SNDRs are respectively, expressed as

(15) $$\begin{array}{c} \gamma_{DF1i}=\min\left(\gamma_{r1i},\gamma_{r2i}\right), \end{array}$$

(16) $$\begin{array}{c} \gamma_{DF2i}=\min\left(\gamma_{r3i},\gamma_{r4i}\right). \end{array}$$

Similar to AF protocol, the opportunistic relay selection scheme is also used; the final SNDR of DF protocol is given by

(17) $$\begin{array}{c} \gamma_{de}=\max_{i\in \left\{1,\ldots ,N\right\}}\left\{\min\left(\gamma_{DF1i},\gamma_{DF2i}\right)\right\}. \end{array}$$

### 3.2. OP

In HSTCN, OP is an important performance measure, which is defined as the probability that the instantaneous SNDR falls below a predefined threshold $x_{0}$. Before deriving the OP of the system, what requires to be considered principally is to get the probability distribution function (PDF) of $\lambda_{1i}$ and $\lambda_{2i}$, respectively.

According to [20], the PDF for $\lambda_{1i}$ is given by

(18) $$\begin{array}{c} f_{\lambda_{1i}}\left(\lambda_{1i}\right)=\sum_{\xi_{1}=0}^{m_{1}-1}\cdots \sum_{\xi_{M=0}}^{m_{1}-1}\Xi \left(M\right)\lambda_{1i}^{\Lambda_{1i}-1}e^{-\Delta_{1i}\lambda_{1i}}, \end{array}$$

where

$$\Xi \left(M\right)\overset{\Delta }{=}\prod_{\tau =1}^{M}\vartheta \left(\xi_{\tau }\right)\alpha_{1i}^{M}\prod_{\upsilon =1}^{M-1}B\left(\sum_{l=1}^{\upsilon }\xi_{l}+\upsilon ,\xi_{\upsilon +1}+1\right),$$

$\Lambda_{1i}\overset{\Delta }{=}\sum_{\tau =1}^{M}\xi_{\tau }+M$, $\vartheta \left(\xi_{\tau }\right)=\frac{{\left(1-m_{\tau }\right)}_{\xi_{\tau }}{\left(-\delta_{\tau }\right)}^{\xi_{\tau }}}{{\left(\xi_{\tau }!\right)}^{2}{\left({\bar{\lambda }}_{1i}\right)}^{\xi_{\tau }+1}}$, $B\left(.,.\right)$ denotes the Beta function [43] and $\Delta_{1i}=\frac{\beta_{1i}-\delta_{1i}}{{\bar{\lambda }}_{1i}}$. ${\bar{\lambda }}_{1i}$ is the average SNR of the $S_{1}$- the *i*-th R channel, $\alpha_{1i}\overset{\Delta }{=}\frac{{\left(\frac{2b_{1i}m_{1i}}{2b_{1i}m_{1}+\Omega_{1i}}\right)}^{m_{1}}}{2b_{1i}}$, $\beta_{1}\overset{\Delta }{=}\frac{1}{2b_{1i}}$, $\delta_{1i}\overset{\Delta }{=}\frac{\Omega_{1}}{2b_{1}\left(2b_{1i}m_{1}+\Omega_{1i}\right)}$, $\Omega_{1i},2b_{1i}$ and $m_{1}\geq 0$ correspond to the average power of the LOS component, the average power of the multi-path component and the fading severity parameter ranging from 0 to *∞*, respectively. ${\left(\cdot \right)}_{q}$ is the Pochhammer symbol.

Furthermore, the PDF of $\lambda_{2i}$ can be uniformly written as

(19) $$\begin{array}{c} f_{\lambda_{2i}}\left(\lambda_{2i}\right)=\sum_{i=1}^{\rho \left(A_{2i}\right)}\sum_{j=1}^{\tau_{i}\left(A_{2i}\right)}\chi_{i,j}\left(A_{2i}\right)\frac{{\bar{\lambda }}_{2i}^{-j}}{\left(j-1\right)!}\lambda_{2i}^{j-1}e^{-\lambda_{2i}/{\bar{\lambda }}_{2i}}, \end{array}$$

where $A_{2i}=\mathrm{diag}\left({\bar{\lambda }}_{21},\ldots ,{\bar{\lambda }}_{2i},\ldots ,{\bar{\lambda }}_{2M}\right)$, $\rho \left(A_{2i}\right)$ is the number of distinct diagonal elements of $A_{l}$, ${\bar{\lambda }}_{21}>{\bar{\lambda }}_{22}>\ldots >{\bar{\lambda }}_{2\langle \rho \left(A_{2i}\right)\rangle }$ are the distinct diagonal elements in decreasing order, $\tau_{i}\left(A_{2i}\right)$ is the multiplicity of ${\bar{\lambda }}_{2i}$, and $\chi_{i,j}\left(A_{2i}\right)$ is the (i,j)-th characteristic coefficient of $A_{2i}$ [44].

#### 3.2.1. OP for AF Protocol

With the help of (8) and (9), the OP of the system at $S_{1}$ and $S_{2}$ form the *i*-th R link are given by (20) and (21), respectively, which are at the top of this page.

(20) $$\begin{array}{c} P_{out1}\left(x_{0}\right)=\left\{\begin{array}{c} \int_{0}^{\frac{D_{1}x_{0}}{1-A_{1}x_{0}}}\mathrm{Pr}\left\{\lambda_{2i}\left(\lambda_{1i}-\lambda_{1i}A_{1}x_{0}-D_{1}x_{0}\right)\leq \lambda_{1i}^{2}B_{1}x_{0}+\lambda_{1i}C_{1}x_{0}+E_{1}x_{0}\right\}f_{\lambda_{1i}}\left(\lambda_{1i}\right)d\lambda_{1i}\\ +\int_{\frac{D_{1}x_{0}}{1-A_{1}x_{0}}}^{\infty }\mathrm{Pr}\left(\lambda_{2i}\leq \frac{\lambda_{1i}^{2}B_{1}x_{0}+\lambda_{1i}C_{1}x_{0}+E_{1}x_{0}}{\lambda_{1i}-\lambda_{1i}A_{1}x_{0}-D_{1}x_{0}}\right)f_{\lambda_{1i}}\left(\lambda_{1i}\right)d\lambda_{1i},x_{0}<\frac{1}{A_{1}}\\ 1,x_{0}\geq \frac{1}{A_{1}}, \end{array}\right. \end{array}$$

(21) $$\begin{array}{c} P_{out2}\left(x_{0}\right)=\left\{\begin{array}{c} \int_{0}^{\frac{D_{2}x_{0}}{1-A_{2}x_{0}}}\mathrm{Pr}\left\{\lambda_{1i}\left(\lambda_{2i}-\lambda_{2i}A_{2i}x_{0}-D_{2i}x_{0}\right)\leq \lambda_{2i}^{2}B_{2}x_{0}+\lambda_{2i}C_{2}x_{0}+E_{2}x_{0}\right\}f_{\lambda_{2i}}\left(\lambda_{2i}\right)d\lambda_{2i}\\ +\int_{\frac{D_{2}x_{0}}{1-A_{2}x_{0}}}^{\infty }\mathrm{Pr}\left\{\lambda_{1i}\leq \frac{\lambda_{2i}^{2}B_{2}x_{0}+\lambda_{2i}C_{2}x_{0}+E_{2}x_{0}}{\lambda_{2i}-\lambda_{2i}A_{2i}x_{0}-D_{2}x_{0}}\right\}f_{\lambda_{2i}}\left(\lambda_{2i}\right)d\lambda_{2i},x_{0}<\frac{1}{A_{2}}\\ 1,x_{0}\geq \frac{1}{A_{2}}. \end{array}\right. \end{array}$$

Substituting (18) and (19) into (20) and (21), with the help of [43] and after some mathematical steps, (20) and (21) are reexpressed as (22) and (23), respectively, which are also at the bottom of this page and at the top of next page, respectively,

(22) Pout1x0=∑ξ1=0m1−1⋯∑ξM=0m1−1ΞMΔ1iΛ1iγΛ1i,Δ1iD1x01−A1x0+∑ξ1=0m1−1⋯∑ξM=0m1−1∑i=1ρA2i∑j=1τiA2i∑s=0Λ1i−1Λ1i−1sΞMχi,jA2iλ¯2i−jj−1!×e−Δ1iX1X1Λ1i−1−sj−1!λ¯2ijs!Δ1i−s−1−2e−J2λ¯2i∑v=0j−1∑t=0v∑m=0v−tvtv−tmj−1!λ¯2ij−vJ1tJ2v−t−mJ3mv!×J3Δ1iλ¯2i+J1t+s−m+12Kt+s−m+12J3λ¯2iΔ1i+J1λ¯2i,x0<1A11,x0≥1A1,

where $X_{1}=\frac{D_{1}x_{0}}{1-A_{1}x_{0}}$, $J_{1}=\frac{B_{1}x_{0}}{1-A_{1}x_{0}}$, $J_{2}=\frac{2X_{1}B_{1}x_{0}+C_{1}x_{0}}{1-A_{1}x_{0}}$, $J_{3}=\frac{X_{1}^{2}B_{1}x_{0}+X_{1}C_{1}x_{0}+E_{1}x_{0}}{1-A_{1}x_{0}}$, $X_{2}=\frac{D_{2}x_{0}}{1-A_{2}x_{0}}$, $J_{4}=\frac{B_{2}x_{0}}{1-A_{2}x_{0}}$, $J_{5}=\frac{2X_{2}B_{2}x_{0}+C_{2}x_{0}}{1-A_{2}x_{0}}$, $J_{6}=\frac{X_{2}^{2}B_{2}x_{0}+X_{2}C_{2}x_{0}+E_{2}x_{0}}{1-A_{2}x_{0}}$, $K_{p+1}\left(\cdot \right)$ is the (p+1)-th-order modified Bessel function of the second kind.

(23) Pout2x0=∑i=1ρA2i∑j=1τiA2iχi,jA2ij−1!γj,D2x01−A2x0λ¯2i+∑ξ1=0m1−1⋯∑ξM=0m1−1∑i=1ρA2i∑j=1τiA2iχi,jA2iΞMλ¯2i−jj−1!∑p=0j−1j−1p×X2j−1−pe−X2λ¯2iΛ1i−1!Δ1iΛ1ip!λ¯2ip+1−2e−J5Δ1i∑q=0Λ1i−1∑r=0q∑w=0q−rΛ1i−1!q!Δ1iΛ1i−qqrq−rwJ4rJ6wJ5q−r−w×1J4Δ1i+λ¯2ir−w+p+12Kr−w+p+12J4Δ1i+μiλ¯2i2,x0<1A21,x0≥1A2.

With the help of (10), the final expression of the OP for AF protocol is given by

(24) $$\begin{array}{c} P_{out-AF}\left(x_{0}\right)={\left[P_{out1}\left(x_{0}\right)+P_{out2}\left(x_{0}\right)-P_{out1}\left(x_{0}\right)P_{out2}\left(x_{0}\right)\right]}^{N}. \end{array}$$

By substituting (22) and (23) into (24), the closed-form expression of the OP for AF protocol is derived.

#### 3.2.2. OP for DF Protocol

From (11), we can get the cumulative distribution function (CDF) for $\gamma_{r1i}$ as

(25) $$\begin{array}{c} F_{r1i}\left(x_{0}\right)=\int_{0}^{\infty }\mathrm{Pr}\left(\lambda_{2i}\leq \frac{\lambda_{1i}F_{1}x_{0}+H_{1}x_{0}}{1-L_{1}x_{0}}\right)f_{\lambda_{1i}}\left(\lambda_{1i}\right)d\lambda_{1i}. \end{array}$$

By substituting (18) and (19) into (25), (25) can be rewritten as (26), which is at the top of this page,

(26) Fr1ix0=∑i=1ρA2i∑j=1τiA2i∑ξ1=0m1−1⋯∑ξM=0m1−1ΞMχi,jA2iλ¯2i−jj−1!j−1!λ¯2ijΛ1i−1!Δ1iΛ1i−e−Y2∑v=0j−1∑s=0vvsj−1!Y2v−sY1sλ¯2ij−vv!s+Λ1i−1!Δ1i+Y1−s−Λ1i,x0<1F11,x0≥1F1,

where $Y_{1}=\frac{F_{1}x_{0}}{1-L_{1}x_{0}}$ and $Y_{2}=\frac{x_{0}}{1-L_{1}x_{0}}$.

With the same method, the CDF for $\gamma_{r2i}$ can be derived as

(27) $$\begin{array}{c} F_{r2i}\left(x_{0}\right)=\left\{\begin{array}{c} \sum_{\xi_{1}=0}^{m_{1}-1}\cdots \sum_{\xi_{M=0}}^{m_{1}-1}\frac{\Xi \left(M\right)}{\Delta_{1i}^{\Lambda_{1i}}}\gamma \left(\Lambda_{1i},\frac{\Delta_{1i}L_{2}x_{0}}{1-F_{2}x_{0}}\right),x_{0}<\frac{1}{F_{2}}\\ 1,x_{0}\geq \frac{1}{F_{2}}. \end{array}\right. \end{array}$$

With the help of (13), we can get the CDF for $\gamma_{r3i}$ as

(28) $$\begin{array}{c} F_{r3i}\left(x_{0}\right)=\int_{0}^{\infty }\mathrm{Pr}\left(\lambda_{1i}\leq \frac{\lambda_{2i}F_{2}x_{0}+H_{2}x_{0}}{1-L_{2}x_{0}}\right)f_{\lambda_{2i}}\left(\lambda_{2i}\right)d\lambda_{2i}. \end{array}$$

By substituting (18) and (19) into (28), (28) is rewritten as (29), which is at the top of next page, where $Y_{3}=\frac{F_{3}x_{0}}{1-L_{3}x_{0}}$ and $Y_{4}=\frac{x_{0}}{1-L_{3}x_{0}}$.

(29) Fr3ix0=∑ξ1=0m1−1⋯∑ξM=0m1−1∑i=1ρA2i∑j=1τiA2iχi,jA2iΞMλ¯2i−jj−1!Λ1i−1!j−1!λ¯2ijΔ1iΛ1i−e−Δ1iY4∑s=0Λ1i−1∑t=0sΛ1i−1!s!stY4t−sY3st+j−1!Δ1iΛ1i−sΔ1iY3+1/λ¯2i,x0<1F31,x0≥1F3,

With the same steps of deriving (26), the CDF for $\gamma_{r4i}$ is expressed as

(30) $$\begin{array}{c} F_{r4i}\left(x_{0}\right)=\left\{\begin{array}{c} \sum_{i=1}^{\rho \left(A_{2i}\right)}\sum_{j=1}^{\tau_{i}\left(A_{2i}\right)}\frac{\chi_{i,j}\left(A_{2i}\right)}{\left(j-1\right)!}\gamma \left(j,\frac{L_{4}x_{0}}{{\bar{\lambda }}_{2i}\left(1-F_{4}x_{0}\right)}\right),x_{0}<\frac{1}{F_{4}}\\ 1,x_{0}\geq \frac{1}{F_{4}}. \end{array}\right. \end{array}$$

Finally, the closed-form expression of the OP for DF protocol is given by

(31) $$\begin{array}{c} P_{out-DF}\left(x_{0}\right)={\left[P_{out-d1i}\left(x_{0}\right)+P_{out-d2i}\left(x_{0}\right)-P_{out-d3i}\left(x_{0}\right)P_{out-d4i}\left(x_{0}\right)\right]}^{N}, \end{array}$$

where

$$\begin{array}{c} P_{out-d1i}\left(x_{0}\right)=F_{r1i}\left(x_{0}\right)+F_{r2i}\left(x_{0}\right)-F_{r1i}\left(x_{0}\right)F_{r2i}\left(x_{0}\right),\\ P_{out-d2i}\left(x_{0}\right)=F_{r3i}\left(x_{0}\right)+F_{r4i}\left(x_{0}\right)-F_{r3i}\left(x_{0}\right)F_{r4i}\left(x_{0}\right). \end{array}$$

By substituting (26), (27), (29) and (30) into (31), the closed-form expression is derived.

### 3.3. The Asymptotic Analysis for OP

To get the impact of HIs level and number of terrestrial relays on the considered network, the asymptotic results are needed. In this subsection, the asymptotic expressions of OP for both forward protocols are obtained at high SNRs. Besides, we set $P_{1}=P_{2}=\mu P_{r}$, where μ>0 and $P_{1}\to \infty$.

For AF protocol, it can be easily seen that the SNDR in (8) and (9) become asymptotically equal to (32) and (33), respectively.

(32) $$\begin{array}{c} \gamma_{1i}^{\infty }=\frac{\lambda_{2i}}{\lambda_{2i}A_{1}+\lambda_{1i}B_{1}}, \end{array}$$

(33) $$\begin{array}{c} \gamma_{2i}^{\infty }=\frac{\lambda_{1i}}{\lambda_{1i}A_{2}+\lambda_{2i}B_{2}}. \end{array}$$

Hence, with the help of (18) and (19), and after some mathematical steps, the asymptotic expressions of OP for AF protocol are given by (34) and (35), respectively, which are at the top of this and the next page, respectively.

(34) $$\begin{array}{c} P_{out1}^{\infty }\left(x_{0}\right)\\ =\sum_{\xi_{1}=0}^{m_{1}-1}\cdots \sum_{\xi_{M}=0}^{m_{1}-1}\Xi \left(M\right)\left[\left(\Lambda_{1i}-1\right)!\Delta_{1i}^{-\Lambda_{1i}}-\sum_{i=1}^{\rho \left(A_{2i}\right)}\sum_{j=1}^{\tau_{i}\left(A_{2i}\right)}\sum_{v=0}^{j-1}\frac{\chi_{i,j}\left(A_{2i}\right)}{\left(j-1\right)!{\bar{\lambda }}_{2i}^{v}}\frac{{\left(B_{1}x_{0}\right)}^{v}{\left(1-A_{1}x_{0}\right)}^{\Lambda_{1i}}\left(\Lambda_{1i}-1+v\right)!}{{\left(\left(1-A_{1}x_{0}\right)\Delta_{1i}+B_{1}x_{0}\right)}^{\Lambda_{1i}+v}}\right], \end{array}$$

(35) $$\begin{array}{c} P_{out2}^{\infty }\left(x_{0}\right)\\ =\sum_{i=1}^{\rho \left(A_{2i}\right)}\sum_{j=1}^{\tau_{i}\left(A_{2i}\right)}\frac{\chi_{i,j}\left(A_{2i}\right){\bar{\lambda }}_{2i}^{-j}}{\left(j-1\right)!}\left[\left(j-1\right)!{\bar{\lambda }}_{2i}^{j}-\sum_{\xi_{1}=0}^{m_{1}-1}\cdots \sum_{\xi_{M}=0}^{m_{1}-1}\sum_{s=0}^{\Lambda_{1i}-1}\frac{\Xi \left(M\right)\left(\Lambda_{1i}-1\right)!{\left(B_{2}x_{0}\right)}^{s}{\left(1-A_{2}x_{0}\right)}^{j}{\bar{\lambda }}_{2i}^{s+j}\left(j-1+s\right)!}{s!\Delta_{1i}^{\Lambda_{1i}-s}{\left(1-A_{2}x_{0}+{\bar{\lambda }}_{2i}\Delta_{1i}B_{2}x_{0}\right)}^{s+j}}\right]. \end{array}$$

For DF protocol, it can be seen easily that (11), (12), (13) and (14) at high SNRs are, respectively, given by

(36) $$\begin{array}{c} \gamma_{r1i}^{\infty }=\frac{\lambda_{2i}}{\lambda_{1i}F_{1}+\lambda_{2i}L_{1}}, \end{array}$$

(37) $$\begin{array}{c} \gamma_{r2i}^{\infty }=\frac{1}{F_{2}}, \end{array}$$

(38) $$\begin{array}{c} \gamma_{r3i}^{\infty }=\frac{\lambda_{1i}}{\lambda_{2i}F_{3}+\lambda_{1i}L_{3}}, \end{array}$$

(39) $$\begin{array}{c} \gamma_{r4i}^{\infty }=\frac{1}{F_{4}}. \end{array}$$

With the help of (18), (19), (36) and (38), then using the same method to obtain (34) and (35), the asymptotic CDF expressions of (36) and (38) can be expressed as (40), (41), respectively, which are at the top of this page and the next page, respectively.

(40) $$\begin{array}{c} F_{r1i}^{\infty }\left(x_{0}\right)\\ =\sum_{\xi_{1}=0}^{m_{1}-1}\cdots \sum_{\xi_{M}=0}^{m_{1}-1}\Xi \left(M\right)\left[\left(\Lambda_{1i}-1\right)!\Delta_{1i}^{-\Lambda_{1i}}-\sum_{i=1}^{\rho \left(A_{2i}\right)}\sum_{j=1}^{\tau_{i}\left(A_{2i}\right)}\sum_{v=0}^{j-1}\frac{\chi_{i,j}\left(A_{2i}\right)}{\left(j-1\right)!{\bar{\lambda }}_{2i}^{v}}\frac{{\left(F_{1}x_{0}\right)}^{v}{\left(1-L_{1}x_{0}\right)}^{\Lambda_{1i}}\left(\Lambda_{1i}-1+v\right)!}{{\left(\left(1-L_{1}x_{0}\right)\Delta_{1i}+F_{1}x_{0}\right)}^{\Lambda_{1i}+v}}\right], \end{array}$$

(41) $$\begin{array}{c} F_{r3i}^{\infty }\left(x_{0}\right)\\ =\sum_{i=1}^{\rho \left(A_{2i}\right)}\sum_{j=1}^{\tau_{i}\left(A_{2i}\right)}\frac{\chi_{i,j}\left(A_{2i}\right){\bar{\lambda }}_{2i}^{-j}}{\left(j-1\right)!}\left[\left(j-1\right)!{\bar{\lambda }}_{2i}^{j}-\sum_{\xi_{1}=0}^{m_{1}-1}\cdots \sum_{\xi_{M}=0}^{m_{1}-1}\sum_{s=0}^{\Lambda_{1i}-1}\frac{\Xi \left(M\right)\left(\Lambda_{1i}-1\right)!{\left(F_{3}x_{0}\right)}^{s}{\left(1-L_{3}x_{0}\right)}^{j}{\bar{\lambda }}_{2i}^{s+j}\left(j-1+s\right)!}{s!\Delta_{1i}^{\Lambda_{1i}-s}{\left(1-L_{3}x_{0}+{\bar{\lambda }}_{2i}\Delta_{1i}F_{3}x_{0}\right)}^{s+j}}\right]. \end{array}$$

From (37) and (39), we know that if these equations are exploited, the asymptotic expressions of DF protocol will not be derived. Hence, we recall the PDF of $\lambda_{1i}$ and $\lambda_{2i}$ at high SNRs to solve this equation.

At high SNRs, the PDF of $\lambda_{1i}$ and $\lambda_{2i}$ are given by, respectively

(42) $$\begin{array}{c} f_{\lambda_{1i}}\left(\lambda_{1i}\right)\approx \frac{\alpha_{1i}^{M}}{{\bar{\lambda }}_{1i}^{M}\left(M-1\right)!}\lambda_{1i}^{M-1}+o\left[\lambda_{1i}^{M-1}\right], \end{array}$$

where $o\left[\cdot \right]$ is the infinitesimal of higher order.

(43) $$\begin{array}{c} f_{\lambda_{2i}}\left(\lambda_{2i}\right)\approx \frac{{\bar{\lambda }}_{2i}^{-M}}{\left(M-1\right)!}\lambda_{2i}^{M-1}+o\left[\lambda_{2i}^{M-1}\right]. \end{array}$$

Then with the help of (12), (14), (42) and (43), the asymptotic CDF expressions for $\gamma_{r2i}$ and $\gamma_{r4i}$ at high SNRs are, respectively, given by

(44) $$\begin{array}{c} F_{r2i}^{\infty }\left(x_{0}\right)=\frac{\alpha_{1i}^{M}}{{\bar{\lambda }}_{1i}^{M}\left(M\right)!}{\left(\frac{L_{2}x_{0}}{1-F_{2}x_{0}}\right)}^{M}, \end{array}$$

(45) $$\begin{array}{c} F_{r4i}^{\infty }\left(x_{0}\right)=\frac{{\bar{\lambda }}_{2i}^{-M}}{\left(M\right)!}{\left(\frac{L_{4}x_{0}}{1-F_{4}x_{0}}\right)}^{M}. \end{array}$$

Then, substituting (34) and (35) into (24), and taking (40), (41), (44) and (45) into (31), the asymptotic expressions of OP for both two forward protocols would be derived, respectively.

### 3.4. The Throughput of the System

It is essential for us to analyze the throughput for the system, especially the mobile user $S_{2}$. According to [31], the definition of throughput for two time slots networks can be expressed as

(46) $$\begin{array}{c} T=\frac{R_{s}}{2}\times \left[1-P_{out-r}\left(x_{0}\right)\right],r\in \left\{AF,DF\right\}. \end{array}$$

By substituting (24) and (31) with analytical and asymptotic OP expressions into (46), the analytical and the asymptotic expressions of throughput are derived. In order to reduce the length of the paper, we do not give the final expressions here.

## 4. Numerical Results

**Figure 2:**
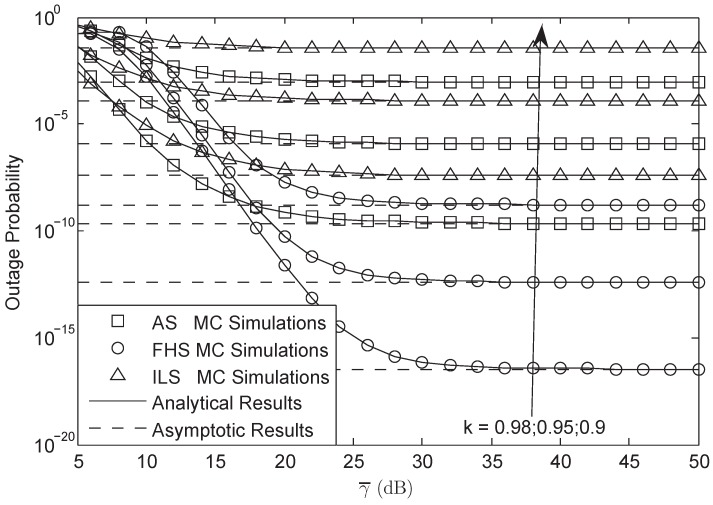
OP of the system for AF protocol versus different $\bar{\gamma }$.

**Figure 3:**
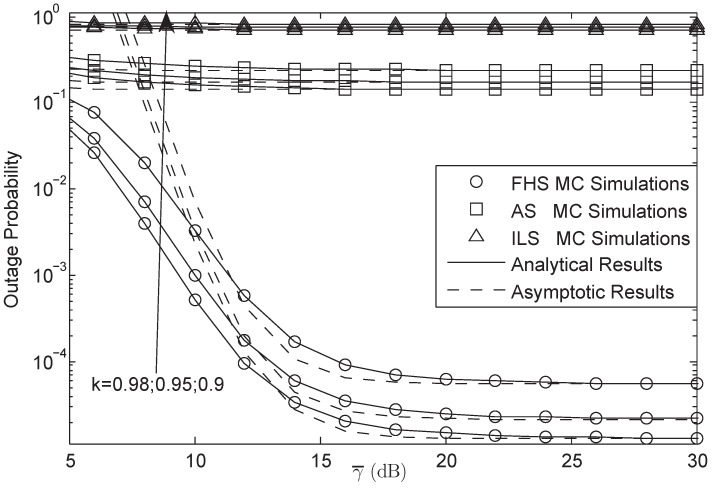
OP of the system for DF protocol versus different $\bar{\gamma }$.

**Figure 4:**
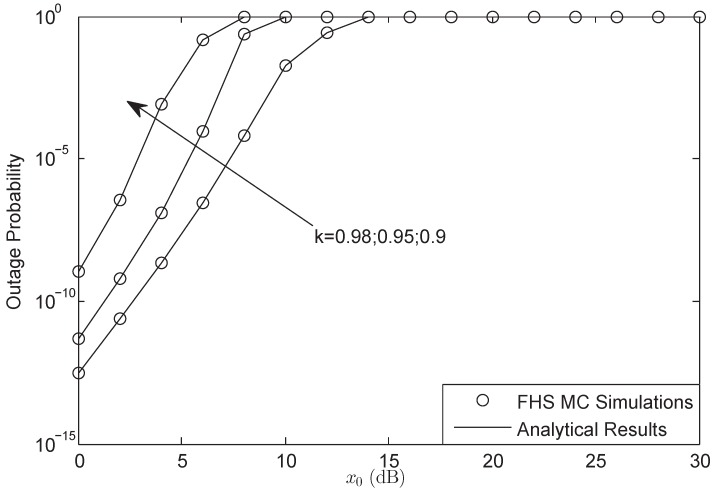
OP of the system for AF protocol versus different $x_{0}$: FHS.

**Figure 5:**
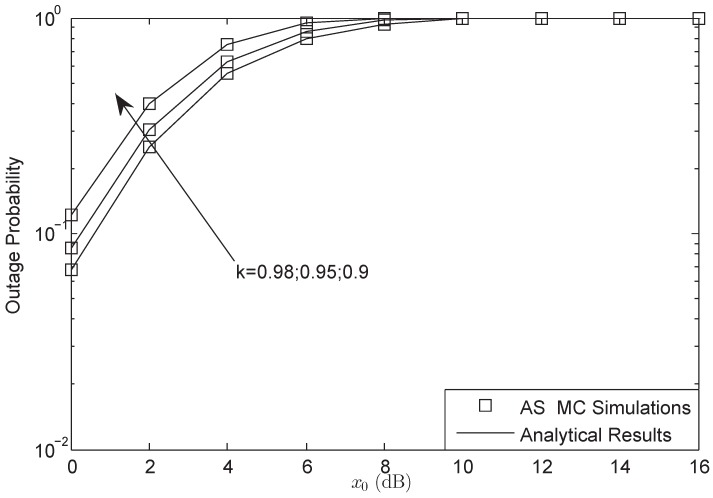
OP of the system for DF protocol versus different $x_{0}$: AS.

**Figure 6:**
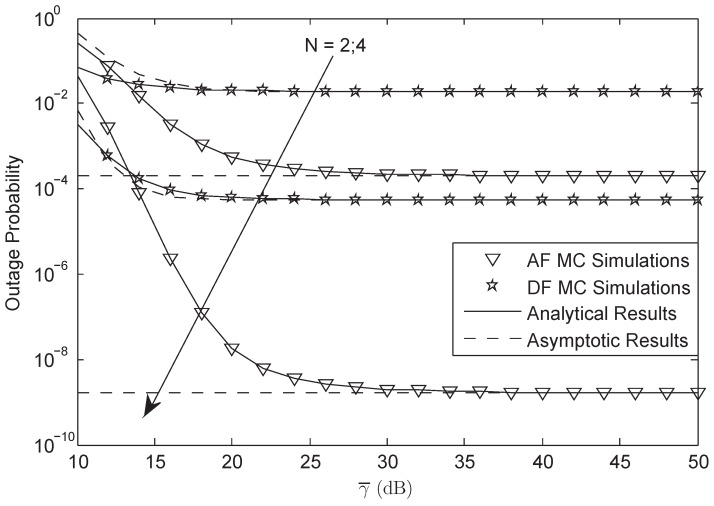
OP of the system versus N=2 and N=4: FHS.

**Figure 7:**
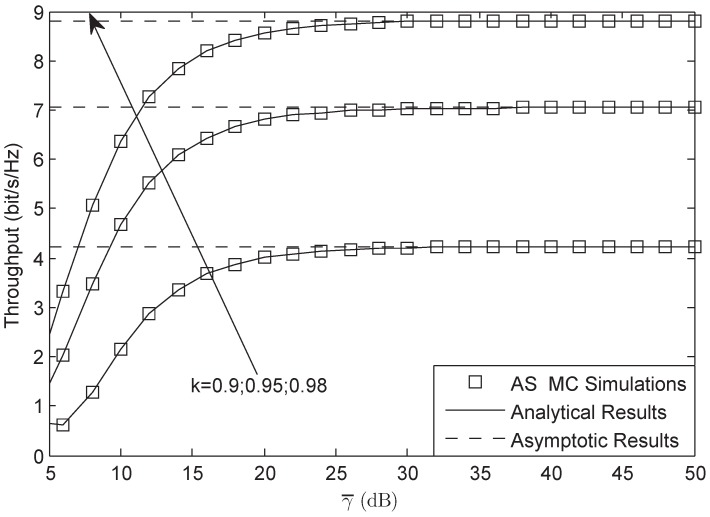
Throughput of the system for AF protocol versus different $\bar{\gamma }$: AS.

**Figure 8:**
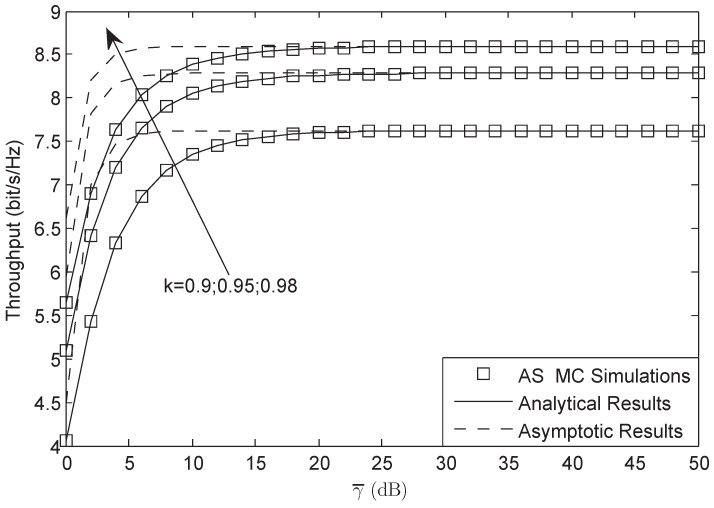
Throughput of the system for DF protocol versus different $\bar{\gamma }$: AS.

In this section, numerical computer simulations are provided to verify the theoretical analysis and show the impacts of key parameters on the system performance. In what follows, we set $P_{1}=P_{2}=\mu P_{r}$, $\delta_{1}^{2}=\delta_{2}^{2}=\delta_{r}^{2}$, ${\bar{\lambda }}_{1i}={\bar{\lambda }}_{2i}=\bar{\gamma }$, $R_{s}=20$ bit/s/Hz, and M=3; Furthermore, we assume that the $S_{1}$-R and $S_{2}$-R link have the same impairment level in Figure 2, Figure 3, Figure 4, Figure 5, Figure 6, Figure 7 and Figure 8, which means that $k_{1}=k_{2}=k_{3}=k$. The parameters for the shadowed-Rician fading channel are located Table 1.

Figure 2 and Figure 3 plot the OP of the system versus different $\bar{\gamma }$ for AF and DF protocols, respectively. From both figures, we first observe that the simulation results are tight across the analytical results versus the entire SNRs, which justify the correctness of the analytical results. Secondly, we can see that the OP of the system will be fixed when the system is under HIs and the SNR is high enough. Thirdly, we also get that the system will have lower OP when the HIs level is larger. Finally, we derive that the OP between AF and DF protocol is different at the same SNR where the OP of AF is lower than that of DF protocol, which implies the advantages of AF protocol in this paper.

Figure 4 and Figure 5 show that the OP of the system versus different $x_{0}$ for AF and DF protocols, respectively. According to the different forward protocols, the predefined threshold $x_{0}$ ranges from 0 dB to 30 dB and 16 dB, respectively. The outage threshold will have a bound when the system is suffering HIs, which has been proved by (24) and (31). When the threshold is larger than the bound, the OP will be always 1. From both figures, we also know that the threshold bound of system is just the function of the HIs level. The larger impairments level is, the larger threshold bound would be obtained. Besides, the bound of DF protocol is lower than that of AF protocol, which is the character of the forward protocol in HIs environment.

Figure 6 depicts OP of the system versus different $\bar{\gamma }$ for different *N*. It indicates that when more terrestrial relays are employed with the system, the system performance will be enhanced. The increasing of the terrestrial relay number will bring a great performance gain to the system.

Figure 7 and Figure 8 illustrate the throughput of the system for both AF and DF protocols, respectively. From both figures, we observe that the throughput is lower than that of the target rate $R_{s}$ for the reason that the system suffers HIs. The results from both figures also suggest that the system will have better performance when the level of the HIs is larger. They also depict that the system will have worse performance when the channel is under heavy shadowed-Rician fading. In addition, we know that the impact of HIs on DF protocol is larger than that of AF protocol in the considered network, which is the character of the considered HIs system.

**Figure 9:**
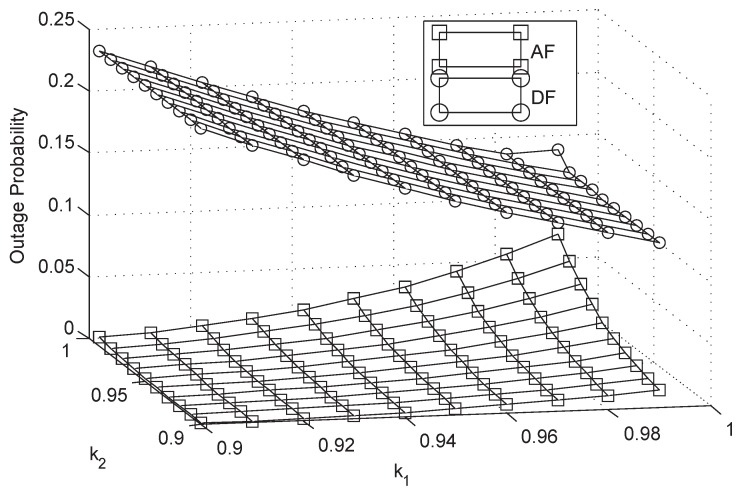
OP of the system for AF and DF protocols versus different hardware impairment level: AS.

Figure 9 plots the OP of the system for AF and DF protocols versus different HIs level in AS. In this figure, we assume that $k_{1}+k_{2}+k_{3}=2.7$ [37]. From the figure, we can observe that the OP of AF protocol is lower than that of DF protocol which has been verified before. Besides, it also suggests that the system will have better system performance when the HIs level of each transmitted node is equal in AF protocol. However, for DF protocol, if we want to have better system performance, the impairments level of terrestrial relay should be smaller.

## 5. Discussion

In this paper, we have investigated the performance of the two-way satellite multiple terrestrial relay networks with HIs and opportunistic relay selection scheme, where both AF and DF protocols have been considered in the system. Firstly, we have obtained the new SNDR of the system with the practical HIs model. Secondly, we have derived the analytical expressions of the OP and throughput for both AF and DF protocols, respectively, from which we can get the fast ways to calculate the effect of HIs on the considered system. Thirdly, to get the effects of HIs on the considered system at high SNRs, the asymptotic expressions of the system performance have been given. The results have shown that the OP and throughput would have a lower bound and a high bound at high SNRs when the system was under HIs. Besides, the larger HIs level is, the larger the bound of the threshold. The larger HIs level is, the larger the bound of the throughput. Fourthly, from the numerical simulations, we found that the system would get better performance when the number of terrestrial relay was larger. Moreover, we have researched the OP versus different HIs level; the results suggested that if we wanted to have better system performance, the HIs level of each node should have been matched according to the forward protocol.

## Figures and Tables

**Table 1:** Channel Parameters.

Shadowing	$m_{1i}$	$b_{1i}$	$\Omega_{1i}$
Frequent heavy shadowing (FHS)	1	0.063	0.0007
Average shadowing (AS)	5	0.251	0.279
Infrequent light shadowing (ILS)	10	0.158	1.29

## Abbreviations

The following abbreviations are used in this manuscript:

HIs	hardware impairments
AF	amplify-and-forward
DF	decode-and-forward
OP	outage probability
SNRs	signal-to-noise ratios
Satcom	satellite communication
LOS	line-of-sight
HSTCN	hybrid satellite-terrestrial cooperative networks
MRC	maximal ratio combining
MRT	maximum ratio transmission
SER	symbol error rate
BF	beamforming
OP	outage probability
PDF	probability distribution function
CDF	cumulative distortion function
FHS	Frequent heavy shadowing
AS	Average shadowing
ILS	Infrequent light shadowing
